# Avoidance of air pollution by home-based high tone therapy in cancer patients

**DOI:** 10.1007/s00508-026-02727-7

**Published:** 2026-03-17

**Authors:** Robert Wakolbinger-Habel, Christof Pabinger, Harald Lothaller, Klaus Hohenstein, Richard Crevenna, Tatjana Paternostro-Sluga

**Affiliations:** 1 Department of Physical and Rehabilitation Medicine (PRM), Vienna Healthcare Group—Clinic Donaustadt, Langobardenstraße 122, 1220 Vienna, Austria; 2 External Lecturer, Medical University of Vienna, Währinger Gürtel 18–20, 1090 Vienna, Austria; 3 Institute for Regenerative Medicine (IRM), Plüddemanngasse 45, Graz, Austria; 4 Telemed Austria, Plüddemanngasse 45, 8010 Graz, Austria; 5 University of Music and Performing Arts Graz, Leonhardstraße 15, 8010, 8010 Graz, Austria; 6 Department of Physical and Rehabilitation Medicine (PRM), Vienna Healthcare Group—Clinic Ottakring, Montleartstraße 37, 1160 Vienna, Austria; 7 Department of Physical Medicine, Rehabilitation and Occupational Medicine, Medical University of Vienna, Währinger Gürtel 18–20, 1090 Vienna, Austria

**Keywords:** HiToP 191 PNP, Home-based electrotherapy, Oncological rehabilitation, Ecological footprint, CO_2_ emissions

## Abstract

**Background:**

Home-based treatments are highly relevant for cancer patients due to their limited access to rehabilitation resources. Therefore, home-based treatments offer several advantages in the oncologic setting. In addition, there is potential to decrease the environmental footprints. No previous study has addressed the co-benefits of home-based electrotherapy. Therefore, the aim of this study was to evaluate the reductions in CO_2_, NOx and particulate emissions using home-based high tone therapy.

**Methods:**

This study was conducted at two supraregional clinics in Vienna, Austria. A total of 65 cancer patients treated for chemotherapy-induced polyneuropathy via home-based high tone therapy over a period of 3 weeks were evaluated. Basic data as well as the distance from the patients’ home to the respective clinic, means of transportation, and treatment adherence were analyzed. Based on this information, the total saved km and emissions of CO_2_, NOx and particulates were calculated.

**Results:**

All patients performed the treatment on at least 15 out of 21 days and 59 patients (91%) even daily. No adverse events were reported and 60% of the patients would have travelled by car and 40% would have used public transport. On average, 129 travel km were saved per patient per treatment week. This corresponds to a saving of 15 kg of CO_2_, 8 g of NOx and 0.4 g of particulates. Assuming an average of 1–3 treatments/week, 1341–4023 km would be saved annually per patient corresponding to a saving of 155–465 kg of CO2, 81–243 g of NOx and 4–13 g of particulates.

**Conclusion:**

Home-based high tone therapy was highly accepted with a high number of treatment sessions among all patient groups. Additionally, strong reductions of emissions of CO_2_, NOx and particulates were demonstrated. It is shown that in Austria alone, home-based therapy could save as many emissions as 77 ha (190 acres) of forest can absorb. Therefore, the home-based treatment may be a promising approach to decrease our environmental footprint.

## Background

Cancer patients have restricted access to rehabilitation resources due to several reasons [1]. For instance, contacts are avoided due to an increased risk of infections but also mobility and activities of daily life are limited due to side effects of oncological treatment. Therefore, home-based treatment options are highly relevant for cancer patients.

Recently, our group published the worldwide first randomized double-blind placebo-controlled study on chemotherapy-induced polyneuropathy using home-based high tone therapy. In the study group, a significant reduction of paresthesia and mental stress due to paresthesia was observed. In an open-label clinical application observation group, several further symptoms (intensity and mental stress due to paresthesia, pain, cramps and intensity of tightness/pressure) were clearly alleviated [2].

Home-based treatments offer several advantages in the oncological setting. First, they are easily accessible as there is no need to travel for each treatment session. In addition, the possible treatment frequency is higher as it can be carried out every day, including weekends. Moreover, as less appointments are necessary, less of the clinic’s resources are occupied, which in turn can be otherwise utilized, thereby improving efficacy.

Furthermore, home-based treatment has a great potential for decreasing environmental footprints due to avoided commuting. In a recent study on telemedicine, digital consultations were offered via videoconferences and significant reductions in CO_2_ emissions were reported [3]. A further study from the USA reported 0.175 metric tons CO_2_ reduction per patient in “teletherapy” but no further information was given on which kind of therapy was offered [4].

To the authors’ knowledge, there is no work on the environmental advances of home-based electrotherapy worldwide. Given the lack of consistent data in the literature on the effects of home-based electrotherapy/high-tone therapy, we conducted this study at two of the largest hospitals in Austria.

Therefore, our study is the first to fully evaluate the reductions in CO_2_, NOx and particulate emissions using home-based high-tone therapy.

## Methods

This two-center study was conducted at the Departments of Physical and Rehabilitation Medicine (PRM) of the Clinic Donaustadt and Clinic Ottakring, Vienna, two supraregional clinics caring not only for patients of Vienna but also for those of surrounding areas of other provinces.

This study was approved by the local Ethics Committee (Ethikkommission der Stadt Wien, EK 24-181-VK) and conducted in full compliance with all applicable local laws as well as in accordance with the Declaration of Helsinki.

Cancer patients treated for chemotherapy-induced polyneuropathy via home-based high tone therapy (HiToP® 191 PNP) were evaluated. The HiToP® 191 PNP (GBO Medizintechnik AG, Rimbach, Germany) is a CE-certified and patented (European Patent 1322379B1) medical device. In detail, the device delivers medium frequency alternating current. The carrier frequency ranges from 4–33 kHz and is then modulated, e.g., 20 times per second, leading to an additional low-frequency current of e.g. 20 Hz. This special procedure is referred as simultaneous frequency amplitude modulation (SimulFAM® (gbo Medizintechnik AG, Rimbach, Germany)).

After a single personal instruction about handling of the device at the respective outpatient facility, the home-based treatment was carried out for 3 weeks with a minimum number of treatment sessions to be completed on 5 out of 7 days per protocol.

Information about travelling distance (in km on the fastest route) from the patients’ home to the respective clinic as well as the means of transportation was collected. The total saved km were calculated by multiplying the distance by 30 (i.e., the total number of journeys per workday to the clinic and back that would have been necessary in the case of outpatient appointments). Calculations per weeks of treatment (assuming 5/7 days) and annually (for 1 treatment/week and for 3 treatments/week) were performed to make our results easier to compare with other studies.

Based on the patients’ diaries, treatment adherence was monitored. In addition, the device itself recorded the hours of stimulation and this information was recorded. Moreover, the patients could reach out to the respective PRM department at any time in the case of questions or issues.

This ensured that feasibility, adherence and safety were effectively monitored.

The calculations of CO_2_, NOx and particulates (PM10) emissions were performed via an accurate and reliable tool by the Austrian Federal Ministry of Climate Action, Environment, Energy, Mobility, Innovation and Technology, in cooperation with the Federal Environmental Ministry (Umweltbundesamt Austria [5]).

Emissions from cars as a means of transportation were calculated based on the average vehicle in Austria. Of 5,185,006 registered cars in 2023 [6], 2,313,859 were powered by petrol, 2,584,985 by diesel, 250,982 by hybrid vehicles and 160,671 by alternative energy sources (electricity, hydrogen or gas). Half of the hybrid vehicles were counted as petrol and half as alternative drives, resulting in shares of 44.63% for petrol, 49.86% for diesel and 5.52% for alternative drives of all registered cars. On the basis of these shares and the figures for total emissions in g/passenger km (pkm) for the three drive types from the source stated above, emission values of 215.88 g CO_2_, 0.474 g NOx and 0.029 g particulates per km were obtained for the average vehicle as a means of transportation.

Emissions for public transport were calculated based on emission values from buses (diesel and electric drive) and rail passenger transport, with each contributing 50%, resulting in emission values of 33.15 g CO_2_, 0.075 g NOx and 0.004 g particulates per km for the public transport mode.

Arithmetic and descriptive statistical methods, in particular measures of central tendency and of dispersion, were used to determine and present the reduction in emissions.

Via regression analysis, potential relationships between treatment sessions and distances to the respective clinic, place of residence, gender, means of transport, and cancer type were analyzed.

For the comparative analyses regarding all new cancer patients in Austria (44,000 patients are registered with newly diagnosed cancer per year in Austria, and according to the results of Burgess [7] and Nielsen [8], approximately 15% of these, i.e., 6600 patients, could be treated with home-based therapy), the relatively high dispersion within the group was addressed with the median instead of the mean value for the extrapolation.

## Results

In this study 65 patients were treated via home-based high tone therapy, 24 patients (37%) had colon cancer, 17 (26%) breast, 5 (8%) pancreatic, 4 (6%) lymphoma, 3 (5%) myeloma, 2 (3%) small intestine, 2 (3%) leukemia, 2 (3%) gastric, 1 (1.5%) ovarian, 1 (1.5%) bladder, 1 (1.5%) cholangiocellular, 1 (1.5%) neuroendocrine, 1 (1.5%) medulloblastoma and 1 (1.5%) lung cancer.

Regarding means of transportation, 60% of the patients travelled by car and 40% used public transport.

None of the patients had legal guardianship, all patients were mentally sound and capable of performing the home-based therapy.

Most patients were able to travel to the respective clinic independently but 7 patients (11%) were driven by a family member or ambulance transportation as they were unfit to travel by themselves.

Table 1 provides age, number of home-based treatment sessions, distance to the clinic as well as adverse events.

**Table 1:** Patients’ characteristics and information about usual commuting

Variable	Category/unit	Result
Gender (*n*, %)	Male	26 (40%)
	Female	39 (60%)
Age (mean ± SD)	Years	63.9 ± 12.9
Place of residence (*n*, %)	Urban	45 (69%)
	Rural	20 (31%)
Type of cancer (*n*, %)	Colorectal	24 (37%)
	Breast	17 (26%)
	Other	24 (37%)
Usual means of transportation (*n*, %)	Public	26 (40%)
	Car	39 (60%)
Treatment sessions (mean ± SD)	Overall	20.4 ± 1.75
	Urban residence	20.7 ± 1.25
	Rural residence	19.8 ± 2.46
	Public transport	20.5 ± 1.63
	Car	20.4 ± 1.84
	Male	20.3 ± 1.95
	Female	20.5 ± 1.62
	Colorectal	19.50 ± 2.7
	Breast	21.0 ± 0.0
	Other	20.8 ± 12.9
Travel distance in km (Md [IQR])	Overall	8.8 [8.1]
	Public transport	5.5 [5.6]
	Car	12.5 [16.2]
	Urban residence	7.0 [4.9]
	Rural residence	19.1 [19.1]

Data are provided as absolute and relative frequency (gender, place of residence, type of cancer, means of transportation), mean ± standard deviation (years of age, number of treatment sessions: overall and subgroups) and median (Md) [interquartile range, IQR] (travel distance in km: overall and subgroups)

All patients performed the treatment on at least 15 out of 21 days and 59 patients (91%) even daily. There were no differences in treatment sessions between males/females, place of residence (urban/rural regions), or means of transport. Patients with colorectal carcinoma had slightly less treatment sessions than patients with breast cancer and other cancer types.

Patients living in rural areas had longer travel distances to the respective clinic than patients living in urban regions. The distances travelled by car were longer than those by public transport.

No adverse events were reported.

Table 2 provides the saved masses of CO_2_, NOx and particulates per patient. The median spared pollutants for an individual patient were 45 kg of CO_2_, 23.4 g of NOx and 1.25 g of particulates during the study or 15 kg of CO_2_, 7.8 g of NOx and 0.4 g of particulates per treatment week. Differentiated by means of transportation, the great majority of emissions were saved by patients who were able to do without their car and only a very small share by those who would have taken public transports. Accordingly, the mean of individual patients was nearly six times higher with respect to NOx and particulates up to 15 times higher with respect to CO_2_ for those with car as compared to those with public transport mode.

**Table 2:** Avoided masses of air pollutant emissions in total and by type of travelling (by car or by public transportation) per individual patient (median [interquartile range])

Pollutant	Unit	Overall (a)	Car	Public transport
CO_2_	g/study	44,687 [7,160-88,726]	80,955 [49,868-154,786]	5,420 [2,735-7,802]
	g/week (5×/w)	14,896 [2,387-29,575]	26,985 [16,623-51,595]	1,807 [912-2,901]
	g/year (1×/w)	154,915 [24,823-307,585]	28,0643 [172,876-536,590]	18,789 [9,481-30,167]
	g/year (3×/w)	464,745 [74,468-922,755]	841,930 [518,629-1,609,770]	56,368 [28,443-90,500]
NOx	g/study	23.4 [14.5-103.2]	68.2 [22.1-177.9]	12.3 [6.2-19.7]
	g/week (5×/w)	7.8 [4.8-34.4]	22.7 [7.4-59.3]	4.1 [2.1-6.6]
	g/year (1×/w)	81.1 [50.3-357.6]	236.3 [76.4-616.6]	42.5 [21.5-68.3]
	g/year (3×/w)	243.4 [150.9-1072.9]	709.0 [229.3-1849.8]	127.5 [64.4-204.8]
PM10	g/study	1.25 [0.77-6.24]	3.64 [1.18-10.75]]	0.65 [0.33-1.05]
	g/week (5×/w)	0.42 [0.26-2.08]	1.21 [0.39-3.58]	0.22 [0.11-0.35]
	g/year (1×/w)	4.33 [2.68-21.62]	12.60 [4.08-37.27]	2.27 [1.14-3.64]
	g/year (3×/w)	12.98 [8.05-64.85]	37.81 [12.23-111.81]	6.80 [3.43-10.92]

(a) assuming 60% of trips by car

Moreover, we investigated the question of whether aspects of mobility influence commitment to treatment. In the regression analysis, the corrected R^2^ was 0.107 (*p* = .048), i.e., 11% of the differences in treatment sessions could be explained by the predictors. In particular, only the type of cancer was significant: Patients with colorectal cancer (*p* = .018) performed 1.2 treatment sessions less than patients with other cancer types. Neither the distance nor the means of transportation nor the place of residence (i.e. urban vs. rural) are predictors of the number of treatment sessions, there are therefore no restrictions on the broad use of home-based therapy in this respect.

Table 3 shows the results for 6600 possible new cancer patients in Austria per year. A reduction of mean 294 tons of CO_2_, 154 kg of NOx and 8 kg of particulates in the intense therapy variant (5 sessions per week over 3 weeks) and 1022 tons of CO_2_, 535 kg of NOx and 29 kg of particulates in the regular therapy variant (2 units per week over 6 months as currently commonly practiced) could be calculated as compared to home therapy, where emissions are net zero. In the scenario of 3 treatments per week over 1 year (inspired by a study on home-based dialysis [9]), 3967 tons of CO_2_, 1606 kg of NOx and 86 kg of particulates would be saved.

**Table 3:** Extrapolation of masses of air pollutants avoided using different scenarios for all of Austria (median-based). Scenario B (regular therapy over half a year with two treatments per week) corresponds to the avoided masses per year with one treatment per week

	CO_2_ (t)	NOx (kg)	Particulates (kg)
*At the hospital*			
A: Intense therapy over 3 weeks (15 sessions)	294	154	8
B: Regular therapy over 6 months (52 sessions)	1022	535	29
C: 1-year therapy 3 times a week (156 sessions)	3967	1606	86
*At home*	*0*	*0*	*0*

## Discussion

To the authors’ knowledge, this is the first study on the environmental data of home-based electrotherapy worldwide.

Our study has shown a noticeable reduction in emissions by home-based electrotherapy for 65 patients at 2 supraregional clinics in Vienna.

In other Austrian regions the distances from patients’ home to the respective clinics are even longer, compared to our study. According to the Austrian Social Insurance [10], the median distance is 14.8 km, i.e., 68% further as compared to the 8.8 km in our study. In addition, most of Austrian regions can be referred to as rural and, therefore, it is likely that an even higher percentage of patients would travel by car. Moreover, in rural regions a higher amount of public transport is carried out via diesel-fueled busses. Therefore, an even stronger reduction emissions can be assumed for other Austrian regions.

The saved emissions were calculated for 15 treatments, reflecting 1 treatment per business day in an outpatient setting over 3 weeks but most of our patients performed 20 treatments at home. Therefore, one could argue that the potential reductions in emissions might be even stronger (by more than 1/3). Even though there were slight differences in the number of treatment sessions between patients with different cancer types, the adherence was high with more than 19 sessions in all subgroups. This indicates high acceptance of the home-based treatment and the patients obtained a high overall dosage of high tone therapy. Moreover, the home-based treatment seems to be safe as no adverse events were reported.

Furthermore, we intended to extrapolate from our results to the possible nationwide ecological effects of home-based high tone therapy. In Austria, 44,000 new cases of cancer are diagnosed every year [11]. According to the results of Burgess et al. [7] and Nielsen et al. [8], approximately 15% of these could be treated with home-based therapy. It is therefore interesting to estimate the possible reduction in emissions in these 15% (6600 patients). We calculated this for two therapy variants: Once for a short, intensive therapy as in our study with at least 5 units per week (= 15 sessions) and a duration of 3 weeks and once for a currently commonly practiced therapy with 2 units per week over a period of 6 months (= 52 sessions).

An impressive reduction of nearly 300 tons of CO_2_ with the intensive therapy variant and more than 1000 tons of CO_2_ with the regular therapy variant is possible as well as up to 535 kg of NOx and 29 kg of particulates. According to the German Ministry of the Environment (Umweltbundesamt) [12], intensive home-based therapy would save 56,000 euros in environmental pollution costs, while regular home-based therapy would save as much as 194,000 euros in the 6600 Austrian cancer patients as compared to standard therapy in the hospital.

To better illustrate these 1000 tons of saved CO_2_ emissions, we provide 2 examples: First, a typical Austrian 4‑person household emits about 1000 kg of CO_2_ for energy consumption annually. Therefore, the amount of CO_2_ saved by home-based high tone therapy would be equivalent to the emissions produced by 1000 households [13]. Second, one hectar (ha) of forest can store about 13 tons of CO_2_ per year. Therefore, the savings of home-based high tone therapy in Austria could be equivalent to the CO_2_ absorption capacity of 77 ha (190 acres) of forest [14].

On the level of individual patients, the above results for the second therapy variant are in line with Dullet et al. [4] regarding the reduction of CO_2_ emissions of approximately 155 kg per patient. Additionally, 81 g of NOx and 4.3 g of particulates would be saved per patient over 52 sessions. In a study on home-based dialysis, the transport-related savings per patient were 0.955 tons CO_2_ per year [9]; however, the calculations were based on 3 journeys per week over 52 weeks. Moreover, exclusively gas-fueled cars were used, and no public transport.

When relating our calculations of saved CO_2_ emissions per person (155 kg in the regular therapy variant), home-based high tone therapy could significantly support the reduction of the individual ecological footprint. The Austrian per capita emissions of CO_2_ are about 8.7 tons per year [15]. In order to reach the goal of limiting global heating to 1.5 °C above preindustrial levels, CO_2_ emissions per capita need to decrease to 2.3 tons annually by 2030, i.e., a fast and strong reduction compared to the current consumption [16]. Therefore, numerous actions are necessary to reach this ambitious goal. Home-based electrotherapy offers a good opportunity for two reasons: first, our study demonstrated a high potential of reductions in emissions via the home-based treatment and second, many people are more receptive for environmentally friendly actions when they do not need to make any sacrifices.

The environmental benefits observed in this study are unlikely to be specific to HiToP alone but could also apply to other therapies that can be delivered in a home-based setting, such as TENS (transcutaneous electrical nerve stimulation); however, as the required empirical data were only available for patients receiving HiToP, our analysis was restricted to this treatment modality.

A limitation concerns our assumption that time saved by avoiding travel does not generate additional emissions. Patients could reallocate this time to other activities, some of which could be associated with energy use or mobility. As we did not collect data on time-use patterns, we were unable to model these potential effects. Therefore, our estimates should be interpreted as reflecting avoided travel-related emissions and may represent an upper bound of net emission savings.

In summary, home-based high tone therapy was highly accepted with a high number of treatment sessions among all patient groups. In addition, our study demonstrates strong reductions of emissions of CO_2_, NOx and particulates. Therefore, the home-based treatment could be a promising approach to decrease our environmental footprint.
